# 1800MHz Microwave Induces p53 and p53-Mediated Caspase-3 Activation Leading to Cell Apoptosis *In Vitro*

**DOI:** 10.1371/journal.pone.0163935

**Published:** 2016-09-30

**Authors:** Fuqiang Xing, Qiuqiang Zhan, Yiduo He, Jiesheng Cui, Sailing He, Guanyu Wang

**Affiliations:** 1 SCNU-ZJU Joint Research Center of Photonics, South China Academy of Advanced Optoelectronics, South China Normal University (SCNU), 510006 Guangzhou, China; 2 Department of Biology, South University of Science and Technology of China (SUSTC), Shenzhen 518055, China; 3 Department of Electromagnetic Engineering, Royal Institute of Technology (KTH), 10044 Stockholm, Sweden; Toho Daigaku, JAPAN

## Abstract

Recent studies have reported that exposure of mammalian cells to microwave radiation may have adverse effects such as induction of cell apoptosis. However, the molecular mechanisms underlying microwave induced mammalian cell apoptosis are not fully understood. Here, we report a novel mechanism: exposure to 1800MHz microwave radiation induces p53-dependent cell apoptosis through cytochrome *c*-mediated caspase-3 activation pathway. We first measured intensity of microwave radiation from several electronic devices with an irradiation detector. Mouse NIH/3T3 and human U-87 MG cells were then used as receivers of 1800MHz electromagnetic radiation (EMR) at a power density of 1209 mW/m^2^. Following EMR exposure, cells were analyzed for viability, intracellular reactive oxygen species (ROS) generation, DNA damage, p53 expression, and caspase-3 activity. Our analysis revealed that EMR exposure significantly decreased viability of NIH/3T3 and U-87 MG cells, and increased caspase-3 activity. ROS burst was observed at 6 h and 48 h in NIH/3T3 cells, while at 3 h in U-87 MG cells. Hoechst 33258 staining and in situ TUNEL assay detected that EMR exposure increased DNA damage, which was significantly restrained in the presence of N-acetyl-L-cysteine (NAC, an antioxidant). Moreover, EMR exposure increased the levels of p53 protein and p53 target gene expression, promoted cytochrome *c* release from mitochondrion, and increased caspase-3 activity. These events were inhibited by pretreatment with NAC, pifithrin-α (a p53 inhibitor) and caspase inhibitor. Collectively, our findings demonstrate, for the first time, that 1800MHz EMR induces apoptosis-related events such as ROS burst and more oxidative DNA damage, which in turn promote p53-dependent caspase-3 activation through release of cytochrome *c* from mitochondrion. These findings thus provide new insights into physiological mechanisms underlying microwave-induced cell apoptosis.

## Introduction

Human exposure to electromagnetic radiation (EMR) has increased dramatically in recent years, due to widespread use of various electronic devices, especially mobile phones. Devices that generate electromagnetic fields include radio or radar station transmitters, power transmission lines, high frequency welders, microwave ovens, and so on. Studies on the biological effects of EMR increase dramatically in recent years, as widespread uses of mobile phones have caused increasing concerns and debates regarding their implications to human health [1, 2]. Although it is still controversial about the risk to human health from EMR exposure, the International Agency for Research on Cancer (IARC) has evaluated human cancer risks from EMR exposure and classified EMR as a possible carcinogen to humans (2B) [3, 4].

Apoptosis is characterized by a number of genetic and biochemical events, including decreased cell viability, chromatin condensation, DNA fragmentation, and caspase activation. The use of mobile phones exposes human organs to frequent EMR. Recent studies have revealed a possible connection between EMR and impaired cell functions [5, 6], including the demonstration of increased apoptosis in human and animal cells exposed to 1800MHz EMR [7, 8]. Although those studies have demonstrated that EMR can induce cell apoptosis, the underlying molecular mechanisms remain largely unknown.

It is known that the nervous system, in particular the brain, is sensitive to EMR and other environmental factors[9]. Previous works have demonstrated that microwave radiation induces neuron apoptosis via the classical mitochondria-dependent caspase-3 pathway [10]. In addition, embryonic stem cells including mouse embryonic NIH/3T3 cells have been reported to be more sensitive to microwave exposure than differentiated cells. Therefore, they have been used frequently in environmental genotoxicity testing [11, 12]. In the present study, we shall use mouse NIH/3T3 and human U-87 MG cells as our model systems.

It has been established that reactive oxygen species (ROS) can damage various cellular compartments, leading to DNA damage, protein oxidation, lipid peroxidation and apoptosis[13–15]. ROS is constantly produced under normal or mildly stressful conditions; and the basal concentration of ROS is usually pro-proliferative. Under severe stresses, excessive ROS is produced, which can damage DNA and proteins. Previous studies suggested that EMR exposure may affect living cells by increasing the ROS level and causing oxidative stresses [16–18]. The tumor suppressor protein p53 is a transcription factor that mediates numerous extrinsic or intrinsic challenges to the cell, playing pivotal roles such as cell cycle arrest, apoptosis induction and DNA repair [19]. Activation of p53 upregulates pro-apoptosis genes; and the consequential apoptosis effectively prevents the accumulation of abnormal cells[20, 21]. In the present study, we focused on the potential roles played by ROS in cell apoptosis mediated by p53 signaling pathway and caused by 1800MHz EMR.

To test our hypothesis that microwave radiation induces cell apoptosis and to identify its biological mechanisms, we first measured the power densities of various electronic devices, and then selected a suitable one for further study. We then subjected NIH/3T3 and U-87 MG cells to microwave radiation with different time duration to measure their corresponding apoptosis. These works also allowed us to select the effective time duration for further investigation of the mechanism. To ensure that microwave exposure had induced cell apoptosis, we checked several indicators of apoptosis, such as DNA damage, release of cytochrome *c* from mitochondria and decrease in cell viability. Furthermore, we measured p53 expressions and caspase-3 activity, in both NIH/3T3 and U-87 MG cells subjected to 1800MHz radiation.

## Materials and Methods

### Reagents and antibodies

2’,7’-Dichlorodihydrofluorescin diacetate (DCFH-DA) and MitoSOX Red were purchased from Invitrogen (Carlsbad, California). The TdT-mediated X-dUTP nick end labeling (TUNEL) assay kit was purchased from Roche (Roche Molecular Biochemicals,Germany). Ac-DEVD-CHO, Z-VAD-FMKand the caspase-3 activity kit were purchased from Beyotime Institute of Biotechnology (Haimen, China). Hoechst 33258and N-Acetyl-L-cysteine (NAC) were obtained from Sigma (St. Louis, Missouri). Cell Counting Kit-8 (CCK-8) and pifithrin-α (PIF-α, p53 inhibitor) was purchased from Dojindo Laboratories (Kumamoto, Japan) and BioVision (Mountain View, CA, USA), respectively. Anti-p53, β-actin, anti-caspase-3, anti-cytochrome *c* antibodies, and all the secondary antibodies were obtained from Cell Signaling Technology (Beverly, MA).

### Cell culture

The Mouse NIH/3T3 (Catalog No. GNM 6) and human U-87 MG (Catalog No. TCHu138) cell lines were purchased from Cellbank of the Chinese Academy of Sciences. Cells were cultured in Dulbecco’s modified Eagle’s medium (Gibco) supplemented with 10% Fetal bovine serum (HyClone), 100 U/ml penicillin and 100 μg/ml streptomycin (Gibco) at 5% CO_2_ and 37°C in a humidified incubator.

### Measurements of microwave intensity

The power densities of various electronic devices were tested by an EMR detector according to a previously reported method[22]. Briefly, a 50MHz~3.5GHz X-Y-Z 3-dimensional EMR potential detector TES-92 was placed close to the surface of various electronic devices and the intensity of microwave was recorded (peak reading of XYZ mode).

The cell exposure system is composed of a microwave generator and transmitting antenna, the antenna is connected to a microwave generator via a flexible coaxial cable and enclosed in a conventional humidified incubator (5% CO_2_, 37°C). Petri dishes are positioned on the top of the antenna; cells were exposed to continuous microwave radiation at 1800MHz 1209 mW/m^2^ for different periods of time (0, 3, 6, 12, 24, 48 h).

### Cell viability and apoptosis assay

Cell Counting Kit-8 (CCK-8, Dojindo Laboratories, Kumamoto, Japan) was used in the current study to evaluate cell viability after various treatments. Optical density (OD450, the absorbance value at 450 nm) was directly read using a 96-well plate reader (imark, Bio-Rad, USA) from each microplate well. The value was normalized to that of the untreated cells from the same plate.

In situ BrdU-Red DNA Fragmentation (TUNEL) assay kit was used to detect DNA fragment. EMR treated cells were washed twice in PBS and added 50μl/well TUNEL reaction mixture, and were then incubated in a humidified atmosphere for 60 min at 37°C in the dark. Hoechst 33258 staining was performed following the previous method [23]. A stock solution of Hoechst 33258 was prepared at a concentration of 1 mg/ml in distilled water and stored at 4°C in the dark for use. EMR treated cells were washed twice in cold PBS and added Hoechst 33258 (final concentration: 10 μg/ml), and were then incubated at room temperature for 30 min in the dark. Cells whose nuclei exhibiting bright fluorescence, fragmented morphology and typical phenomena of nuclear condensation, were counted as apoptotic cells. Cells exhibiting diffusely fluorescent nuclei were classified as alive and viable. Fluorescence images of the cells were examined with a × 20 objective under a fluorescence microscope (Olympus IX71, Olympus Optical Co. Ltd, Tokyo, Japan).

### Detection of intracellular ROS generation

Assessment of intracellular ROS changes in cells during apoptosis was performed using fluorescence probes H_2_DCFDA staining. A cell density of 10^5^/ml was placed in each 35-mm culture plate (Thermo Fisher Scientific). After exposure, cells were preloaded with 10 μM DCFH-DA in phenol red-free culture medium for 30 min, then cells were detached using 0.25% trypsin (Gibco) and transferred to a 4-way cuvette (Hongjun Optics Co., China). Following digestion, the intensity of fluorescence was analyzed by a fluorescence spectrophotometer (F-2500, Hitachi) with excitation at 488 nm and emission at 525 nm. Each experiment was repeated three times.

MitoSOX Red was used to detect mitochondrial ROS production, cells were seeded at a density of 10^5^/ml in glass bottom culture dishes (NEST, 801001). After exposure for 6 hours, cells were preloaded with 5 μM MitoSOX Red in phenol red-free culture medium for 30 min. Then cells were visualized by using two-photon fluoresence microscopy (Olympus FV1000 MPE). The wavelength of femtosecond pulse laser for two-photon excitation was 780 nm.

### Caspase-3 enzymatic activities

The activity of caspase-3 was determined using the caspase-3 activity kit, based on the ability of caspase-3 to change acetyl-Asp-Glu-Val-Asp p-nitroanilide (Ac-DEVD-pNA) into a yellow formazan product p-nitroaniline (pNA). Caspase inhibitor Z-VAD-FMK (100 μM) and caspase-3-like inhibitor Ac-DEVD-CHO (100 μM) were used according to the manufacturer's instructions (Beyotime, Haimen, China). Lysates were centrifuged at 12,000 g for 10 min, and protein concentrations were determined by Bradford protein assay. Cellular extracts (30 μg) were incubated in a 96-well microtitre plate with 20 ng Ac-DEVD-pNA for 4 hours at 37°C. OD405 values were read with a 96-well plate reader. An increase in OD405 indicated the activation of caspase-3.

### Protein extraction and western blot analysis

Detection of protein expression by western blot analysis was performed as described previously [24]. After different exposure treatments, cells were washed twice in cold PBS and lysed on ice with ice-cold lysis buffer (50 mM Tris HCl pH 8.0, 150 mM NaCl, 1% TritonX-100, 100 μg/ml PMSF) for 1 hour. The lysates were centrifuged (12.000g, 5 min, 4°C), followed by protein concentration determination. Equivalent cellular protein (30 μg each lane) were subjected to SDS-Polyacrylamide Gel Electrophoresis (SDS-PAGE) on 12% gel and transferred to a nitrocellulose membrane (Bio-Rad). The membranes with proteins were blocked with 5% skim milk for 0.5 hour and then incubated with primary antibodies at 4°C overnight followed by incubation with fluorescent secondary antibodies for 2 hours at room temperature. Dilution of primary and secondary antibody was 1:1000. Finally, the membranes were scanned on Bio-Rad's ChemiDoc XRS+ system with Image Lab image acquisition and analysis software. The intensity of the western blot signals was quantitated using ImageJ software, and the densitometry analyses are normalized, presented as the ratio protein/β-actin protein.

### Real-time reverse transcription-PCR

Total RNA was isolated by standard trizol protocol and then converted into cDNA. The cDNA was amplified by real-time reverse transcription PCR (RT-PCR) using specific primers to quantify the expression of Bax, PUMA and GLS2, which are all p53 target genes. The primer sequences are presented in S1 Table. All qPCR reactions were performed using the CFX96 real time PCR detection system (Bio-Rad) with the following thermal cycle conditions: 95°C for 2 min, 39 cycles of (95°C for 5s, 60°C for 30 s), followed by a melt curve from 65°C to 95°C with an increment of 0.5°C per cycle. The level of inner loading controls were used to normalize expression of p53 target genes: GAPDH for the detection of Bax and PUMA expression in mouse NIH/3T3 cells; 36B4 (which encodes a ribosomal protein) for the detection of GLS2 and Bax expression in human U-87 MG cells.

### Statistical analysis

Results were expressed as the mean-together with standard deviation (SD). Statistical analysis was performed with Student’s paired *t*-test using the statistical software SPSS, version10.0 (SPSS, Chicago). Differences were considered statistically significant at *P*<0.05 throughout the work. All charts were drawn using the software Origin, version 8.0.

## Results

### Sorting out suitable power density of antenna for cell exposure system

**Fig 1:**
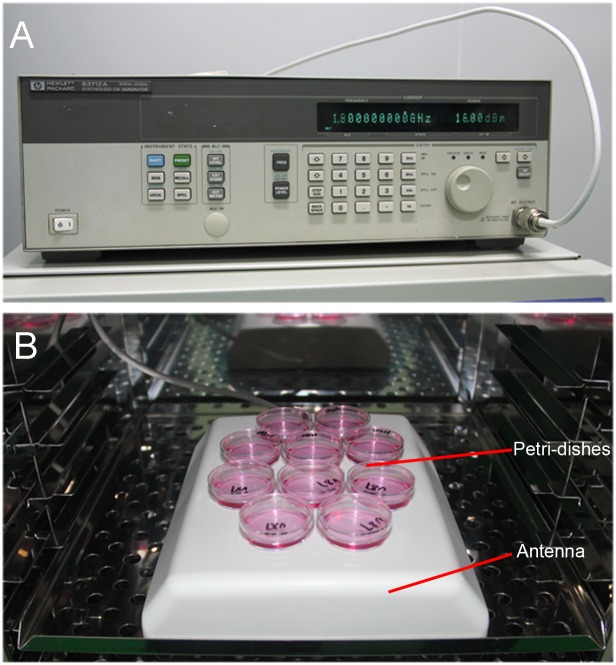
The microwave exposure system. (A) 1800MHz microwave generator. (B) Antenna in an incubator. The antenna was connected to a microwave generator via a flexible coaxial cable and enclosed in a humidified incubator. Petri dishes were positioned on the top of the antenna and cells in petri dishes were exposed to continuous microwave radiation at 1800MHz 1209 mW/m^2^.

In consideration of the fact that power density of electronic devices used in our daily life, especially for cell phones, are constantly changing, we set up a fixed exposure system to assess the effects of EMR on cell physiology (Fig 1A and 1B). With the assistance of an EMR detector (the peak reading of XYZ mode), we tested the changes of microwave radiation power density near the surface of various electronic devices (Table 1 and S1 Fig).

**Table 1:** Intensity test of microwave radiation for various electronic devices (peak reading XYZ mode).

Electronic device	Group	Mean ± SEM (mW/m2)
Antenna (1800MHz)	Sham	0.0001 +0.0002
	Radiation	1209 +268.6081
Cell phone (dialing mode)	A1	9212 +332.5136
	A2	8566 +329.4257
	A3	11330 +227.42910
	A4	26.67 +5.0441
	A5	2774 +315.0132
	A6	29.68 +7.2571
Cell phone (standby mode)	A1	0.2341 +0.1586
	A2	7.425 +0.73179
	A3	5.969 +0.7452
	A4	0.4996 +0.0503
	A5	0.0035 +0.0031
	A6	0.4941 +0.0953
Microwave oven	B1	4119 +70.8731
	B2	4819 +82.1969
Personal computer	C1	5.876 +2.4871
	C2	9.071 +1.8437
Wireless router(WIFI)	D1	31.33 +6.8891
	D2	630.3 +64.3795

A1-D2 denotes electronic devices with different brand

Radiation power density of cell phones (for both dialing and standby modes), microwave oven, personal computer and wireless router were monitored respectively (S1 Fig). We found that cell phone’s power density varies from 26 to 11330 mW/m^2^ during the dialing period; while it varies from 0.0035 to 7.425 mW/m^2^ during the standby period. Notably, the values of personal computer radiation were all below 10 mW/m^2^; wireless routers were a little higher; microwave ovens were much higher, reaching approximately 4500 mW/m^2^. We set 1209 mW/m^2^ as the peak reading of XYZ radiation of antenna at 1800MHz. As expected, the power density of antenna radiation used for this research was higher than the minimum value of cell phones (dialing time and standby time), personal computer and wireless router, but smaller than the maximum value of cell phones (dialing time) and microwave oven (working mode). Hence, these data indicate that 1209 mW/m^2^, the power density of antenna radiation at 1800MHz, can be used to detect the potential mechanisms of EMR induced cell apoptosis in this research.

### 1800MHz microwave induces caspase-3 activation and decrease in cell viability

**Fig 2:**
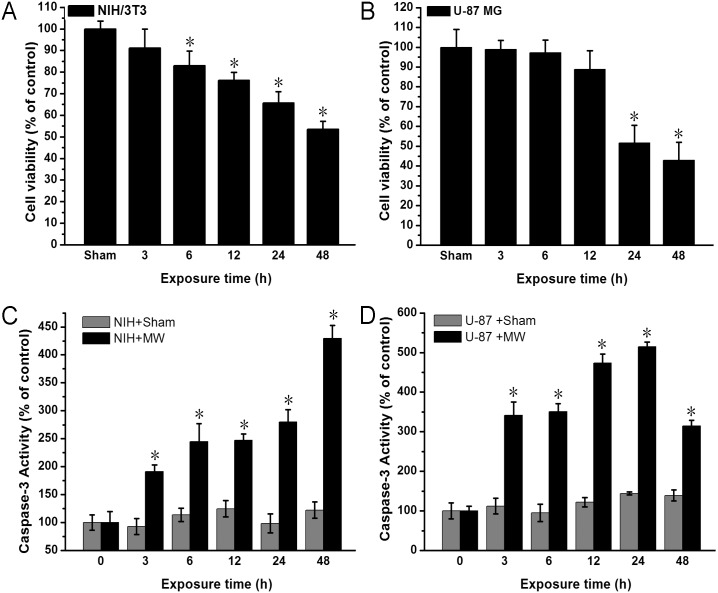
1800 MHz microwave induces caspase-3 activation and decrease in cell viability. (A, B) Levels of cell viability after 1800MHz microwave radiation for the indicated time in NIH/3T3 and U-87 MG cells. Cell Counting Kit-8 (CCK-8, Dojindo Laboratories, Kumamoto, Japan) was used to evaluate cell viability after various treatments. (C, D) Levels of caspase-3 activity after 1800MHz microwave radiation for the indicated time in NIH/3T3and U-87 MG cells. After microwave radiation, cells were harvested, and caspase-3 activity was measured by the caspase-3 activity kit. MW, microwave. Data are the mean ± S.D. (n = 3; *P < 0.05 vs. sham group.)

To test whether 1800MHz microwave with power density strength at 1209 mW/m^2^ induces apoptosis in NIH/3T3 and U-87 MG cells, we determined the cell viability and caspase-3 activity after 1800MHz microwave irradiation. We found that the viability of NIH/3T3 cells decreased gradually from 100% to 55% over 48 hours during 1800MHz microwave irradiation (Fig 2A). Similarly, the viability of U-87 MG cells decreased slightly over 12 hours then rapidly from 24 hour to 48 hours (Fig 2B). We further measured caspase-3 activity after 1800MHz microwave irradiation. Compared with the sham group of NIH/3T3 cells, caspase-3 activity increased gradually over 24 hours, followed by an obvious escalation at 48 hours (Fig 2C). For U-87 MG cells, caspase-3 activity increased rapidly during the first three hours, and then maintained a gradual increase to 24 hours, then decreased (Fig 2D). In summary, these data indicate that 1800MHz microwave irradiation induces caspase-3 dependent apoptosis in NIH/3T3 and U-87 MG cells.

### 1800MHz microwave-induced oxidative DNA damage

**Fig 3:**
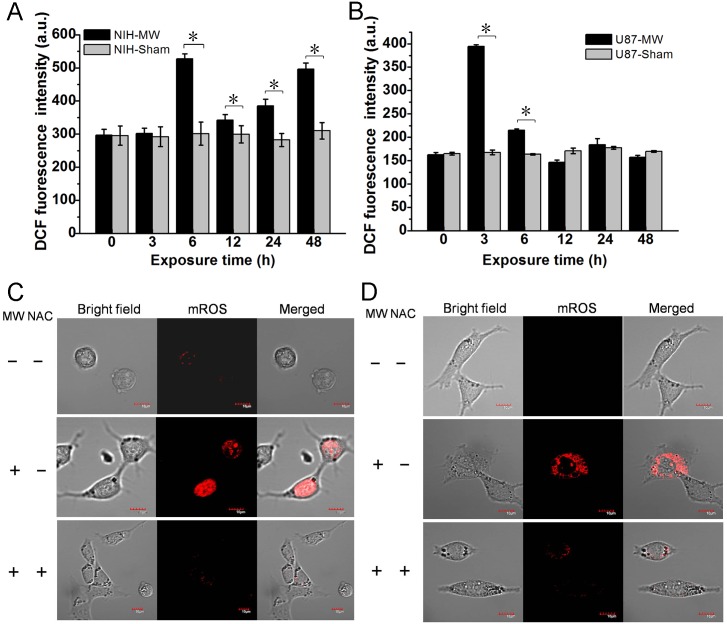
Exposure to 1800MHz microwave induces ROS production in NIH/3T3 and U-87 MG cells. (A, B) Intracellular ROS production was measured by using DCFH-DA after 1800MHz microwave radiation for the indicated time in NIH/3T3 and U-87 MG cells. (C, D) detection of mitochondrial ROS production by using MitoSOX Red after 1800MHz microwave exposure for 6 hours in NIH/3T3 and U-87 MG cells. The imaging experiments were implemented on a two-photon laser scanning microscope system. DCF, 2’,7’-Dichlorodihydrofluorescin diacetate; mROS, mitochondrial reactive oxygen species; MW, microwave; NAC, N-acetyl-L-cysteine.Scale bars: 10 μm. Data represent mean ± S.D. (n = 3; *P < 0.05 vs. sham group.)

**Fig 4:**
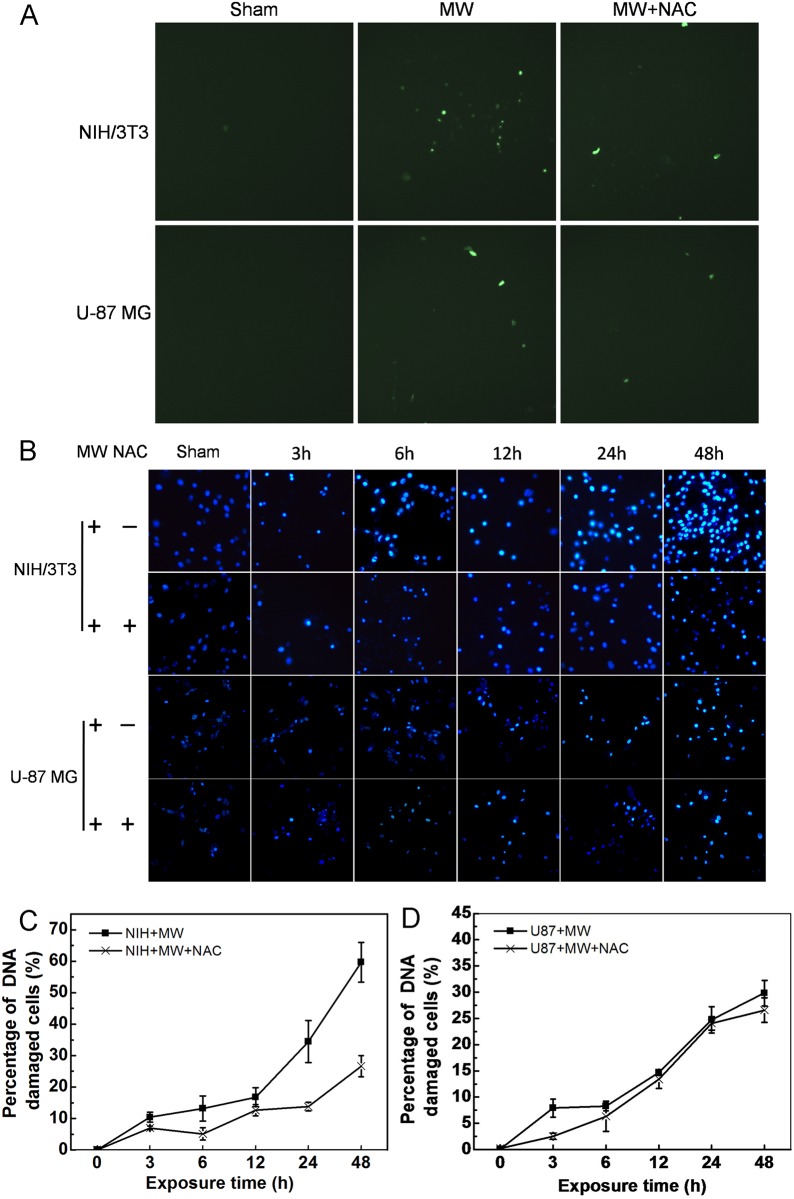
Exposure to 1800MHz microwave induces oxidative DNA damage in NIH/3T3 and U-87 MG cells. (A) In situ TUNEL detection of DNA fragmentation. (B) Hoechst 33258 staining assessment of nuclear condensation in NIH/3T3 and U-87 MG cells. Fluorescent images of DNA damaged cells were visualized under an Olympus fluorescent microscope (20×). (C, D) Quantitative analysis of DNA damages in NIH/3T3 and U87-MG cells stained by Hoechst 33258 shown in (B). MW, microwave; NAC, N-acetyl-L-cysteine. Data represent mean ± S.D. (n = 3; *P < 0.05 vs. sham group.)

To assess whether 1800MHz microwave irradiation can induce ROS generation in NIH/3T3 and U-87 MG cells, we measured intracellular ROS production by using a 2’,7’-dichlorofluorescin diacetate fluorescence probe. NIH/3T3 and U-87 MG cells were irradiated with 1800MHz microwave for different time intervals (0, 3, 6, 12, 24 and 48 hours). We found that, compared with the sham group, 1800MHz microwave irradiation led to fluorescence intensity changes fluctuating greatly over 48 hours in NIH/3T3 cells but only a rapid increase at 3 hours in U-87 MG cells (Fig 3A and 3B). We further detected whether 1800MHz microwave irradiation can induce mitochondrial ROS production by using MitoSOX Red probe on a two-photon laser scanning microscope system (Olympus FV1000 MPE). Our results showed that 1800MHz microwave exposure of NIH/3T3 and U-87 MG cells for 6 hours led to mitochondrial ROS production, which was inhibited by broad-spectrum antioxidant NAC (Fig 3C and 3D). Because nuclear condensation and DNA fragmentation are also hallmarks of cell apoptosis, we then determined whether 1800MHz microwave can induce nuclear condensation and DNA fragmentation by Hoechst 33258 staining and in situ TUNEL assay, respectively. Hoechst 33258 dye is a fluorescent nucleic acid stain for detecting nuclear condensation (Fig 4B and S2 Fig). TUNEL enables highly sensitive detection of apoptosis of single cells. Cells undergoing apoptosis were preferentially labeled by the TUNEL reaction (Fig 4A). To further confirm hypothesis that ROS generation is required for 1800MHz microwave induced DNA damage, NIH/3T3 and U-87 MG cells were pre-treated with the NAC and exposed continuously to EMR for different time intervals (0, 3, 6, 12, 24 and 48 hours). We found that the DNA damage increased NIH/3T3 cells more than two times than the increase of U-87 MG cells, after treatment with 1800MHz microwave (Fig 4C and 4D). These results indicate that 1800MHz microwave exposure of NIH/3T3 and U-87 MG cells cause DNA fragmentation and nuclear condensation. Interestingly, increases in DNA damage were significantly inhibited by NAC in NIH/3T3 cells, but only slightly in U-87 MG cells, supporting the hypothesis that ROS generation is required for DNA injury and cell apoptosis induced by 1800MHz microwave irradiation.

### 1800MHz microwave-induced ROS production upregulates p53 expression

**Fig 5:**
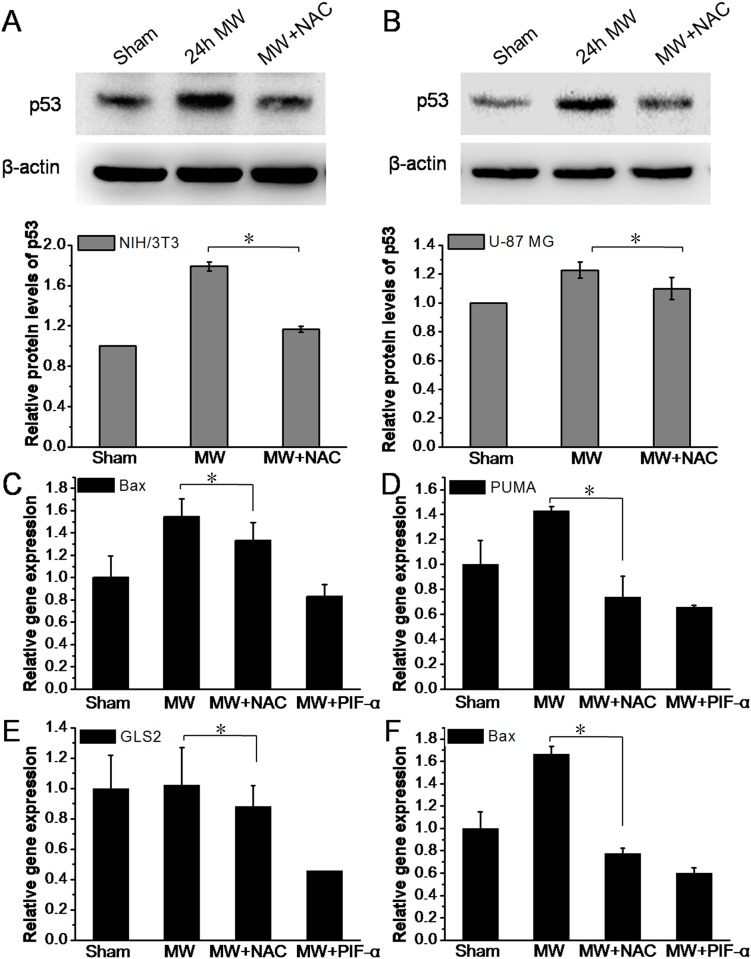
ROS upregulates p53 expression during 1800MHz microwave-induced cell apoptosis. (A, B) ROS induces p53 expression upon 1800MHz microwave radiation for 24 hours in NIH/3T3 and U-87 MG cells. Cells were pretreated with or without NAC and exposed to 1800MHz. The representative western blot assay of p53 exposed to 1800MHz microwave in the presence of NAC in mouse NIH/3T3 and human U-87 MG cells, respectively. (C, D) Detection of the expression of Bax and PUMA, respectively, in mouse NIH/3T3 cells. (E,F) Detection of the expression of GLS2 and Bax, respectively, in human U-87 MG cells. The level of loading controls was used to normalize the expression of p53 target genes: Bax, PUMA, and GLS2. MW, microwave; NAC, N-acetyl-L-cysteine; PIF-α, pifithrin-α. All the data in these figures are presented as mean ± S.D. (n = 3; *P < 0.05 vs. indicated group.)

We then explored the mechanisms of p53 mediated regulation of cell apoptosis induced by 1800MHz microwave. After the irradiation, cells were harvested for protein and mRNA extraction to detect the level of p53 changes and the expression of genes targeted by p53. Western blotting analysis showed that the level of p53 significantly increased upon 1800MHz microwave irradiation relative to the sham group. But this was suppressed by incubation the cells with antioxidant NAC and this suppression effect of p53 expression by NAC was more notable in NIH/3T3 than in U-87 MG cells (Fig 5A and 5B). Moreover, real-time RT-PCR showed that 1800MHz microwave irradiation enhanced expression of p53 target genes: Bax, PUMA and GLS2. These enhanced expressions were significantly inhibited by antioxidant NAC and p53 inhibitor PIF-α (Fig 5C–5F). Together, these results suggested that ROS is involved in 1800MHz microwave-induced upregulation of p53 in cell apoptosis.

### Involvement of ROS and p53 in cytochrome *c*-mediated caspase-3 activation during 1800MHz microwave-induced cell apoptosis

**Fig 6:**
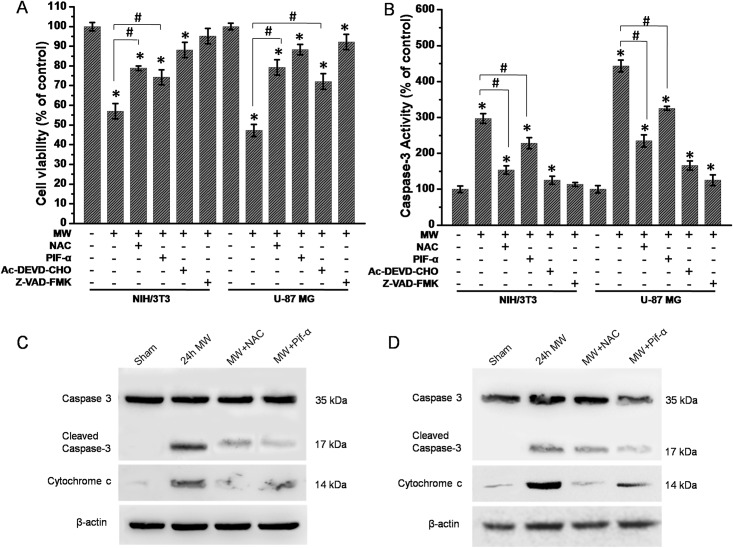
ROS and p53 involve in cytochrome *c*-mediated caspase-3 activation during 1800MHz microwave-induced cell apoptosis. (A, B) Effects of NAC, PIF-α, Ac-DEVD-CHO and Z-VAD-FMK on cell viability and caspase-3 activity after 1800MHz microwave radiation for 24 hours in NIH/3T3 and U-87 MG cells. Cells were pretreated with treatments (NAC, PIF-α, Ac-DEVD-CHO and Z-VAD-FMK) for 20 min and then exposed to 1800MHz microwave or sham treatment for 24 hours. After microwave radiation, cells were harvested and levels of cell viability and caspase-3 activity were measured in NIH/3T3 and U-87 MG cells. Cell Counting Kit-8 (CCK-8, Dojindo Laboratories, Kumamoto, Japan) was used to evaluate cell viability after various treatments. Caspase-3 activity was measured by the caspase-3 activity kit. (C, D) Western blotting analysis of caspase-3 activation and cytochrome *c* release from mitochondria to cytoplasm. Cells were pretreated with or without NAC and PIF-α for 20 min before exposure to 1800MHz microwave. After microwave radiation for 24 hours, cells were harvested and measured protein levels of caspase-3. The cytoplasm proteins were extracted using a mitochondria/cytoplasm fractionation kit followed by western blotting analysis of cytochrome *c* in cytoplasm. MW, microwave; NAC, N-acetyl-L-cysteine. PIF-α, pifithrin-α. All the data in these figures are presented as mean ± S.D. (n = 3; *P < 0.05 vs. control group; #P < 0.05 indicated group.)

Since caspase-3 activation and cytochrome *c* release from mitochondrion are key events in cell apoptosis [25, 26], our results showed that in the presence of Ac-DEVD-CHO (caspase-3 inhibitor) or Z-VAD-FMK (broad-spectrum caspase inhibitor) prevented 1800MHz microwave-induced increase of caspase-3 activity and the consequential decrease of cell viability (Fig 6A and 6B). We also assessed whether or not cell death induced by ROS and p53 upregulation was through cytochrome c-mediated caspase-3 activation pathway. NAC or PIF-α were incubated with NIH/3T3 and U-87 MG cells, respectively. These cells were then subjected to cells subjecting to 1800MHz microwave irradiation. We found that incubation with NAC or PIF-α had strongly blocked the decrease of cell viability and the activation of caspase-3 induced by 1800MHz microwave in NIH/3T3 cells and U-87 MG cells, respectively (Fig 6A and 6B).

We then performed western blotting analysis to assess cytochrome *c* release from mitochondria to cytoplasm. Our results showed that NAC and PIF-α can inhibit the increase in protein levels of caspase-3 and cytochrome *c* in NIH/3T3 cells and U-87 MG cytoplasm (Fig 6C and 6D). Together, these data suggest that ROS and p53 are required for cytochrome *c*-mediated caspase-3 activation induced by 1800MHz microwave in NIH/3T3 and U-87 MG cells.

## Discussion

Numerous studies have been performed to assess human health risks and biological consequences of exposure to microwave radiation, which generated a large bulk of data[27–33]. These data however seem to be contradictory, probably due to different sensitivities of the biological systems to EMR exposure and different parameters of electromagnetic fields. Both factors influence their interactions between EMR and living cells. For instance, single or multiple radiofrequency radiation exposure did not elicit oxidative stress in human breast epithelial MCF10A cells [34]. However, other studies indicated that oxidative stress caused by exposure to Wi-Fi or mobile phone EMR may significantly affect female and male reproductive systems[35]. EMR can also affect gene expression and modulate a wide range of biological processes such as cell-cycle control, apoptosis and autophagy [33, 36, 37]. The present study provides evidences that exposure of NIH/3T3 and U-87 MG cells to EMR at 1800MHz, with a specific power density at approximately 1209 mW/m^2^, induced apoptosis in each cell being investigated (Figs 2, 4 and 6).

Previous studies demonstrated that 1800MHz microwave exposure can induce a dose-dependent or time-dependent ROS generation and DNA damage in fibroblasts cell line [13, 38]. In our experiments, we observed a fluctuating ROS generation in NIH/3T3 cells and transient increase at 3 hours after EMR in U-87 MG cells (Fig 3A and 3B), this may largely due to antioxidant enzymes including superoxide dismutase, glutathione peroxidase, and catalase working together to defend the cells against toxic ROS. Since 1800MHz microwave can also directly induce DNA damage which will further promote p53 activation[39], we speculate that ROS generation and DNA damage may have a synergistic effect in regulating 1800MHz microwave induced p53 activation and cell apoptosis. De et al. found that mobile phones enhance mitochondrial ROS generation and stimulates DNA fragmentation[6]. But it remains to be determined if ROS is responsible for DNA damage, as well as possible pathways through which ROS can affect apoptosis. Here, we observed that 1800MHz microwave irradiation induces ROS generation, which causes DNA damages in NIH/3T3 and U-87 MG cells (Fig 4). Additionally, since 1800MHz microwave irradiation-induced DNA damages were not totally blocked by NAC in our experiment, it is possible that 1800MHz microwave directly induces DNA damage. Furthermore, exposure to intensive electromagnetic fields generated by devices such as radar and mobile phone transmitters can lead to electrical shock and even thermal effects. Many current researchers are also interested in the possible "non-thermal" effects. Previous reports suggested that mobile phone radiation usually does not cause heating but modulates signal transduction pathways and induces cellular stress response [40–42]. In the present study, the dishes and antenna were placed in a humidified incubator (37°C, 5% CO_2_, 95% humidity) to ensure constant environmental conditions for studying the mechanisms of cell apoptosis induced by microwave (Fig 1).

Some conditions in the present study, for example the 48h constant exposure to EMR, seem not to be physiologically relevant. This might be true ten years ago. But with the fast development of wireless and IT industries nowadays, more and more electronic products emerge and more and more people suffer from prolonged EMR due to using these products. For cell phone alone, people use it more frequently, relying on it for communication, education, and e-commerce; it is replacing the laptop. Some people keep the phone near the brain (under the pillow); or near the reproductive system (in a trouser pocket). It is thus common to have a nearly constant exposure to GSM 900/1800MHz cell phone signal in the “on” mode (exposed) or in the ‘‘stand-by” mode (sham) for a long time, approaching or even exceeding 48 hours. To mimic these physiological conditions, we have put NIH/3T3 (embryo fibroblast) and U-87 MG (brain glioblastoma) cells under either exposure or sham (stand-by) conditions for up to 48 hours. Due to facility restrictions, we have used continuous microwaves but not the more realistic pulsed microwaves, which represent one limitation of our work.

Although p53 protein has been implicated to play roles in a variety of cellular processes, its role in cell apoptosis induced by microwave irradiation is still unknown. Here, we found that p53 and p53 target gene induced by 1800MHz microwave irradiation was inhibited by NAC and p53 inhibitor PIF-α pretreatment, suggesting that ROS is involved in regulating the expression of p53 (Fig 5). Besides ROS, many other signals can also activate p53 [43]. Some studies have demonstrated that p53 is extremely sensitive to even low levels of DNA damage, thus promoting DNA repair directly by upregulating the expression of DNA repair genes [44]. In the present study, since 1800MHz microwave irradiation-induced p53 activation was partially blocked by NAC, it is reasonable to speculate that ROS burst and DNA damage together may be involved in upregulating p53 expression during 1800MHz microwave irradiation induced apoptosis in NIH/3T3 and U-87 MG cells. Hussain et al. found that p53-mediated apoptosis may activates redox-controlling genes, resulting in ROS production [45]. In the present study, we confirmed the hypothesis that an oxidative stress induced by 1800MHz microwave irradiation plays a vital role in regulating p53-dependent caspase-3 activation. Caspase-3 is cysteine proteases with well-established functions in promoting apoptosis[46–48]. It is well known that caspase-3 can be activated by proapoptotic molecules such as cytochrome *c* released from mitochondria[49, 50]. Mitochondria are a major source of ROS, mainly at the level of complexes I and III of the respiratory chain [51]. In the present experiment, our results suggested that EMR-induced the release of cytochrome *c* and caspase-3 activation had been blocked by NAC or PIF-α (Fig 6), it is thus safe to say that 1800MHz microwave irradiation induces mitochondrial ROS and the release of cytoplasmic cytochrome *c* and in turn, activates caspase-3 in NIH/3T3 and U-87 MG cells.

**Fig 7:**
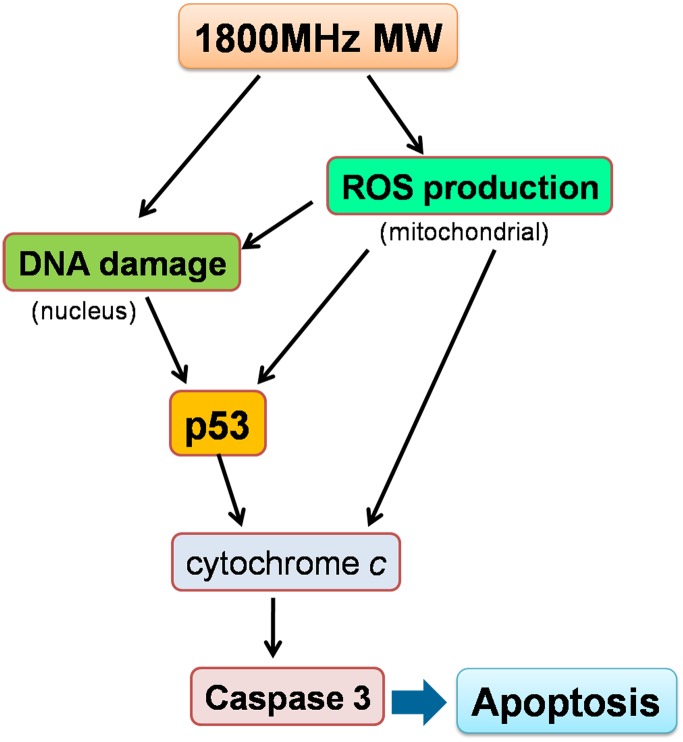
Schematic representation of p53 activation of caspase-3 signaling pathway in 1800MHz microwave induced cell apoptosis. 1800MHz microwave activates p53 signaling pathway through enhancing ROS production and DNA damage, p53 further upregulates cytochrome *c*-mediated caspase-3 activation that leads to cell apoptosis. MW, microwave; NAC, N-acetyl-L-cysteine; ROS, reactive oxygen species.

In conclusion, we report here, for the first time, that exposure of NIH/3T3 and U-87 MG cells to 1800MHz microwave radiation induces ROS production and DNA damage, which in turn up-regulates p53-dependent caspase-3 activation through cytochrome *c*. These events all promote cell apoptosis (Fig 7). Despite these novel findings, it remains unclear how radiation is perceived and how the signal is rapidly transduced into the cells. It is thus important to address these challenges in the future.

## Supporting Information

S1 Fig Determine the intensity of microwave radiation on the surface of various electronic devices. The power densities of antenna,cell phone,microwave oven,personal computer andwireless router were measured by using a 50MHz~3.5GHz X-Y-Z 3-dimensional EMR potential detector TES-92 (peak reading of XYZ mode) on the surface of electronic devices.
(TIF)

S2 Fig Confocal microscopic detection of 1800MHz microwave-induced DNA damage. Hoechst 33258 dye (a fluorescent nucleic acid stain) was used fordetecting nuclear condensation after NIH/3T3 and U-87 MG cells were irradiated with 1800MHz microwave for 48 hours. Cells were visualized by using two-photon fluoresence microscopy (Olympus FV1000 MPE). The wavelength of 730 nm was used for femtosecond pulse laser for two-photon excitation of Hoechst 33258 dye. Arrows indicate nuclei exhibiting bright fluorescence and the fragmented nuclear morphology.
(TIF)

S1 Table primers used in present study. (DOCX)
