# Supplementary figures and images for: 1800MHz Microwave Induces p53 and p53-Mediated Caspase-3 Activation Leading to Cell Apoptosis In Vitro
Source: PLoS One. 2016 Sep 30;11(9):e0163935. doi: 10.1371/journal.pone.0163935 (PMC5045209; doi:10.1371/journal.pone.0163935)

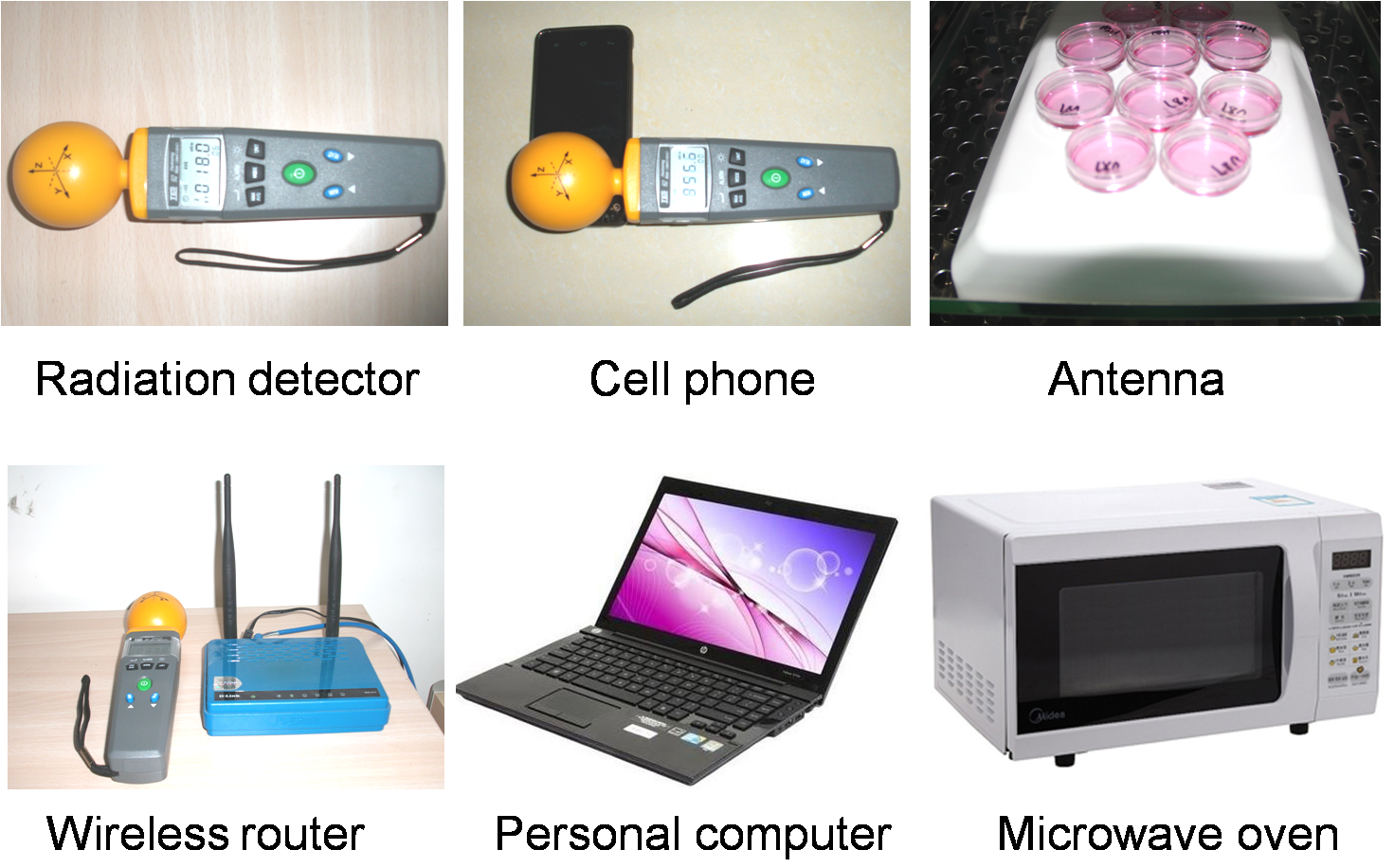

Supplement: S1 Fig — The power densities of antenna,cell phone,microwave oven,personal computer andwireless router were measured by using a 50MHz~3.5GHz X-Y-Z 3-dimensional EMR potential detector TES-92 (peak reading of XYZ mode) on the surface of electronic devices. (TIF) [file pone.0163935.s001.tif]

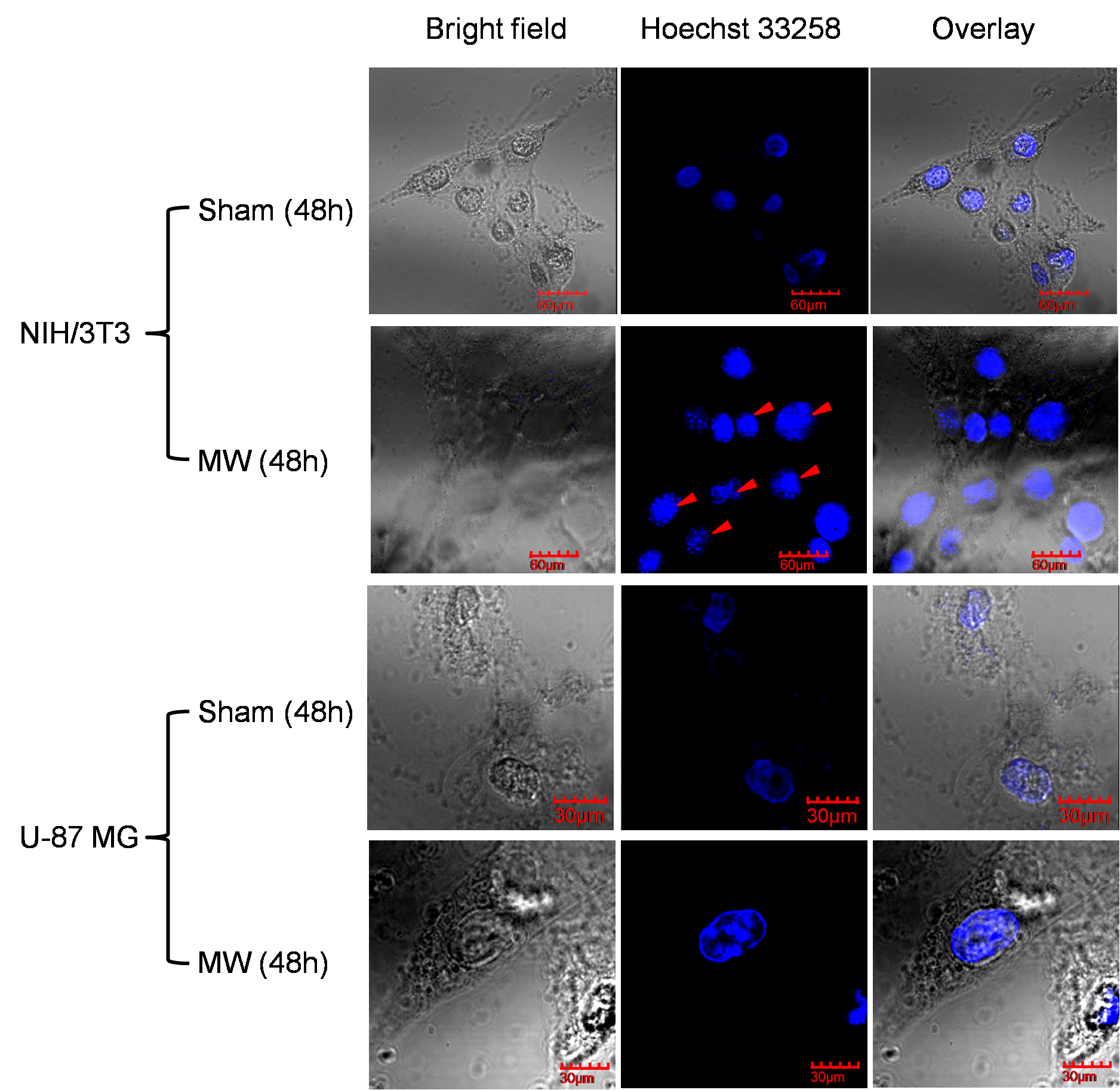

Supplement: S2 Fig — Hoechst 33258 dye (a fluorescent nucleic acid stain) was used fordetecting nuclear condensation after NIH/3T3 and U-87 MG cells were irradiated with 1800MHz microwave for 48 hours. Cells were visualized by using two-photon fluoresence microscopy (Olympus FV1000 MPE). The wavelength of 730 nm was used for femtosecond pulse laser for two-photon excitation of Hoechst 33258 dye. Arrows indicate nuclei exhibiting bright fluorescence and the fragmented nuclear morphology. (TIF) [file pone.0163935.s002.tif]
