# Supplementary material for: 1800MHz Microwave Induces p53 and p53-Mediated Caspase-3 Activation Leading to Cell Apoptosis In Vitro
Source: PLoS One. 2016 Sep 30;11(9):e0163935. doi: 10.1371/journal.pone.0163935 (PMC5045209; doi:10.1371/journal.pone.0163935)
Supplement: S1 Table — (DOCX) [file pone.0163935.s003.docx]

S1 Table: primers used in present study

| Primer | Species | Forward | Reverse |
| --- | --- | --- | --- |
| GAPDH | Mouse | 5'-CCACTCCTCCACCTTTGAC-3' | 5'-ACCCTGTTGCTGTAGCCA-3' |
| Bax | Mouse, Human | 5'-CATGTTTTCTGACGGCAACTTC-3' | 5'-AGGGCCTTGAGCACCAGTTT-3' |
| PUMA | Mouse | 5’-CCTGGAGGGTCCTGTACAATCT-3’ | 5’-GCACCTAATTGGGCTCCATCT-3’ |
| 36B4 | Human | 5’-AGATTCGGGATATGCTGTTG-3’ | 5’-ACATCACTCAGAATTTCAATGG-3’ |
| GLS2 | Human | 5’-TGCCTATAGTGGCGATGTCTCA-3’ | 5’-GTTCCATATCCATGGCTGACAA-3’ |
